# Biogenesis of extracellular vesicles in protozoan parasites: The ESCRT complex in the trafficking fast lane?

**DOI:** 10.1371/journal.ppat.1011140

**Published:** 2023-02-23

**Authors:** Abel Cruz Camacho, Daniel Alfandari, Ewa Kozela, Neta Regev-Rudzki

**Affiliations:** Department of Biomolecular Sciences, Faculty of Biochemistry, Weizmann Institute of Science, Rehovot, Israel; Institut Pasteur, FRANCE

## Abstract

Extracellular vesicles (EVs) provide a central mechanism of cell–cell communication. While EVs are found in most organisms, their pathogenesis-promoting roles in parasites are of particular interest given the potential for medical insight and consequential therapeutic intervention. Yet, a key feature of EVs in human parasitic protozoa remains elusive: their mechanisms of biogenesis. Here, we survey the current knowledge on the biogenesis pathways of EVs secreted by the four main clades of human parasitic protozoa: apicomplexans, trypanosomatids, flagellates, and amoebae. In particular, we shine a light on findings pertaining to the Endosomal Sorting Complex Required for Transport (ESCRT) machinery, as in mammals it plays important roles in EV biogenesis. This review highlights the diversity in EV biogenesis in protozoa, as well as the related involvement of the ESCRT system in these unique organisms.

## I. EV biogenesis in parasitic protozoa

Parasitic protozoa are responsible for a range of diseases in humans. Notable species include *Plasmodium falciparum*, responsible for the deadly malaria disease [1] (*Apicomplexa* phylum), *Trypanosoma brucei*, *Trypanosoma cruzi*, and *Leishmania* spp., responsible for African sleeping sickness, Chagas disease, and leishmaniasis, respectively [2,3], (*Kinetoplastea* phylum, syn. *Kinetoplastida*), *Trichomonas vaginalis* (*Metamonada* phylum), the most prevalent nonviral sexually transmitted human pathogen [4], and *Acanthamoeba castellanii*, a prevalent opportunistic disease [5] (*Amoebozoa* phylum). These single-celled pathogens’ capacity to cause disease in humans and other organisms is dependent on their abilities to invade tissues and cells, coordinate their actions, and manipulate host defenses [6–8]. Studies in recent years have implicated extracellular vesicles (EVs) as mediators of these parasitic activities.

EVs are cell-derived, membrane-enclosed vesicles that transport a variety of cargo components, such as proteins, nucleic acids, and metabolites [7–9]. This robust delivery system enables cell–cell communication by mediating diverse physiological and pathological processes in all kingdoms throughout evolution, from bacteria to humans [10]. EV biogenesis occurs either through the release of exosomes originated in intraluminal vesicles (ILVs) contained within multivesicular bodies (MVBs), upon the fusion of MVBs with a cell’s plasma membrane [11,12], or through direct plasma membrane budding of microvesicles (also called ectosomes) from the cell surface [9,13].

It has been demonstrated that parasitic pathogens, including *P*. *falciparum*, *T*. *brucei*, and *Giardia intestinalis*, produce EVs laden with proteins [6], coding and noncoding RNAs [14–18], and/or DNA [19–21] during infections. They do so either autonomously, by using their own machinery [22–24], or possibly by exploiting their host’s system, just like viruses do [25–27]. Despite the growing consensus as to the importance of EVs in protozoa pathogenesis, our understanding of the mechanisms of EV biogenesis in human parasitic protozoa is still in its infancy, with many questions remaining unanswered. With an eye to advancing this understudied aspect of parasite pathogenesis, in this review, we summarize the findings to date on the pathways involved in EV production in the major human parasitic protozoa.

We will mainly focus on the Endosomal Sorting Complex Required for Transport (ESCRT) system, a key player in the biogenesis of EVs [13]. The ESCRT system mediates several other fundamental cellular processes, including plasma membrane remodeling and maintenance during protein trafficking [28,29], cell organelle compartmentalization (e.g., lysosomes, the nucleus, autophagosomes) [30], sorting of ubiquitinated proteins into ILVs for lysosomal degradation [31], fission and repair of damaged cell membranes [32,33], cell cytokinesis [34,35], and viral egress [36–38]. Composed of approximately 30 proteins, the mammalian ESCRT is a conserved machinery that is localized to endocytic compartments [13]. The ESCRT proteins assemble into five subcomplexes: ESCRT-0, -I, -II, -III, and the ESCRT machinery-associated proteins Alix/Bro1 and the AAA ATPase Vps4 [13].

To generate EVs, ESCRT’s subcomplexes perform a sequence of tasks that lead first to the production of ILVs and then to the generation of exo- or ectosomal EVs [39]. Briefly, ESCRT-0 sequesters ubiquitinated protein cargo, ESCRT-I, -II, and -III induce ILV budding, while the Vps4 enzymatic subcomplex regulates membrane scission [31,40–42]. Each complex comprises specific subunits and domains (e.g., ubiquitin-binding), adaptors, and affiliated proteins (e.g., Tsg101 and Alix in certain exosomes) [43]. These subunits determine their respective unit’s particular roles in the process, the nature of the protein cargo loaded [44], and, eventually, the EV cargo’s destination [37].

Studies into the evolution of the ESCRT system identified numerous homologs of the ESCRT families I, II, III, and associated proteins in a wide variety of protozoa, including *Plasmodium* [22,45], *Trypanosoma* [23,45,46], *Leishmania* [45], *Giardia* [45], and *Entamoeba* [47–51]. It was found that the ESCRT proteins are conserved, suggesting a potentially fully functional system in many of these organisms. This insight highlights the need for further studies concentrating on the specific functions and roles of the ESCRT proteins in parasitic protozoa.

## II. EV functions and biogenesis in *Apicomplexa*: *Plasmodium falciparum* and *Toxoplasma gondii*

Consisting of some 6,000 species [52], apicomplexan parasites are among the most prevalent and morbidity-causing pathogens worldwide, responsible for severe diseases in millions of humans and animals each year [52]. Some of its most known genera that infect humans are *Plasmodium* (haemosporidans), *Toxoplasma* (eucoccidians), *Babesia* (piroplasms), and *Cryptosporidium* (cryptosporidians). Being intracellular obligate parasites, apicomplexans constantly face the hostile environment of the host, which poses unique challenges for signaling and communication both, among the parasite population and within the host [7,53–55].

One of the methods by which apicomplexans manipulate host cells and evade the immune system is the secretion of EVs with different cargo components, which are eventually taken up by various target cells [7,56]. *Apicomplexa*-derived EVs are involved in a multitude of processes, including in transferring virulence factors [18,20,57–59], easing parasite invasion into host cells [58], and modifying the host immune response [18,20,59–62]. Nonetheless, EV biogenesis in apicomplexan parasites has been scarcely studied, and the ESCRT system has been implicated in EV biogenesis only in *P*. *falciparum* [22], the causative agent of the most deadly form of malaria disease [1].

Studies have identified a wide range of active cargo components, such as genomic DNA [19,20], RNA [18,57,59,61], host and parasitic proteins [58,63], glycans [64], and lipids [65,66], in *P*. *falciparum*-derived EVs. Moreover, the cargo composition in the EVs changes with the parasite’s developmental stage, suggesting active cargo sorting into the EVs [20]. *P*. *falciparum*-derived EVs have been shown to advance several pathogenesis-promoting processes in the human host. First, they allow the parasites to communicate among themselves via the exchange of active cargo, even when enclosed inside *P*. *falciparum*-infected human red blood cells (RBCs) [19,61]. In particular, it was demonstrated that *P*. *falciparum* EVs help promote this parasite’s sexual stage development (gametocytogenesis), an essential process for transmission [19,61,67]. Second, parasites use them to manipulate the host’s immune response [18,20,59,61]. Third, they aid in promoting vascular changes and endothelial cell activation [68] and permeability [57], factors that contribute to the establishment of cerebral malaria, the most severe neurological complication of *P*. *falciparum* malaria. Lastly, *P*. *falciparum-*derived EVs prime naïve RBCs for parasite invasion [58]. This feat is achieved by the transfer of assembled and functional 20S proteasome complexes [58], which, upon uptake by naïve human RBCs, alter their cytoskeletal integrity [58]. Similarly, *Plasmodium vivax*, the most widely distributed human malaria parasite, was also shown to produce EVs [69] and manipulate the host’s NF-κB signaling to promote cytoadherence [62].

*Toxoplasma*, the causative agent of toxoplasmosis [70,71], was also shown to secrete EVs. In particular, EVs secreted from *T*. *gondii-*infected human foreskin fibroblasts [72] or dendritic cells [73] exhibit a unique profile of protein and nucleic acids that starkly differs from that of EVs derived from uninfected cells [72,73]. *T*. *gondii*-derived EVs have been reported to modulate the murine macrophage immune response by the induction of IL-10, TNF-α, and iNOS, probably by delivering miRNA to these host cells [60,74]. In addition, the secretion of the inflammatory cytokines IL-12, IFN-γ, and TNF-α, mediated through the JNK pathway, was demonstrated in murine macrophages stimulated with *T*. *gondii* EVs [75]. Interestingly, BALB/c mice inoculated with *T*.*gondii*-derived EVs showed humoral and cellular immune responses as well as a prolonged survival time [74,76], protecting the mice against acute parasite infection. These results may suggest that EVs could serve as vaccine candidates against toxoplasmosis [76].

While accumulating evidence points to the contribution of parasite-derived EVs to malaria and toxoplasmosis pathogenesis, less is known about the underlying mechanisms of EV biogenesis in these organisms and in *Apicomplexa* in general. A study of the phylogenetics of the ESCRT system revealed a lack of the full ESCRT machinery in *Plasmodium*, particularly of the ESCRT-0, -I, and -II subcomplexes [45]. Yet, in silico predictions demonstrated that *P*. *falciparum* possesses at least two putative proteins from the ESCRT-III complex: Vps2 and Vps32/Snf7 [45,77]. Furthermore, an accessory protein to the ESCRT-III complex, the Vps4 ATPase homologue (PfVps4), was found in the cytoplasm of the parasite during its trophozoite blood stage, detected by anti-Vps4 antibodies [78]. When transfected into COS cells, this homologue retained its function in MVB formation, hinting toward the existence of functional ESCRT machinery that mediates the production of MVBs in *P*. *falciparum* [78].

**Fig 1:**
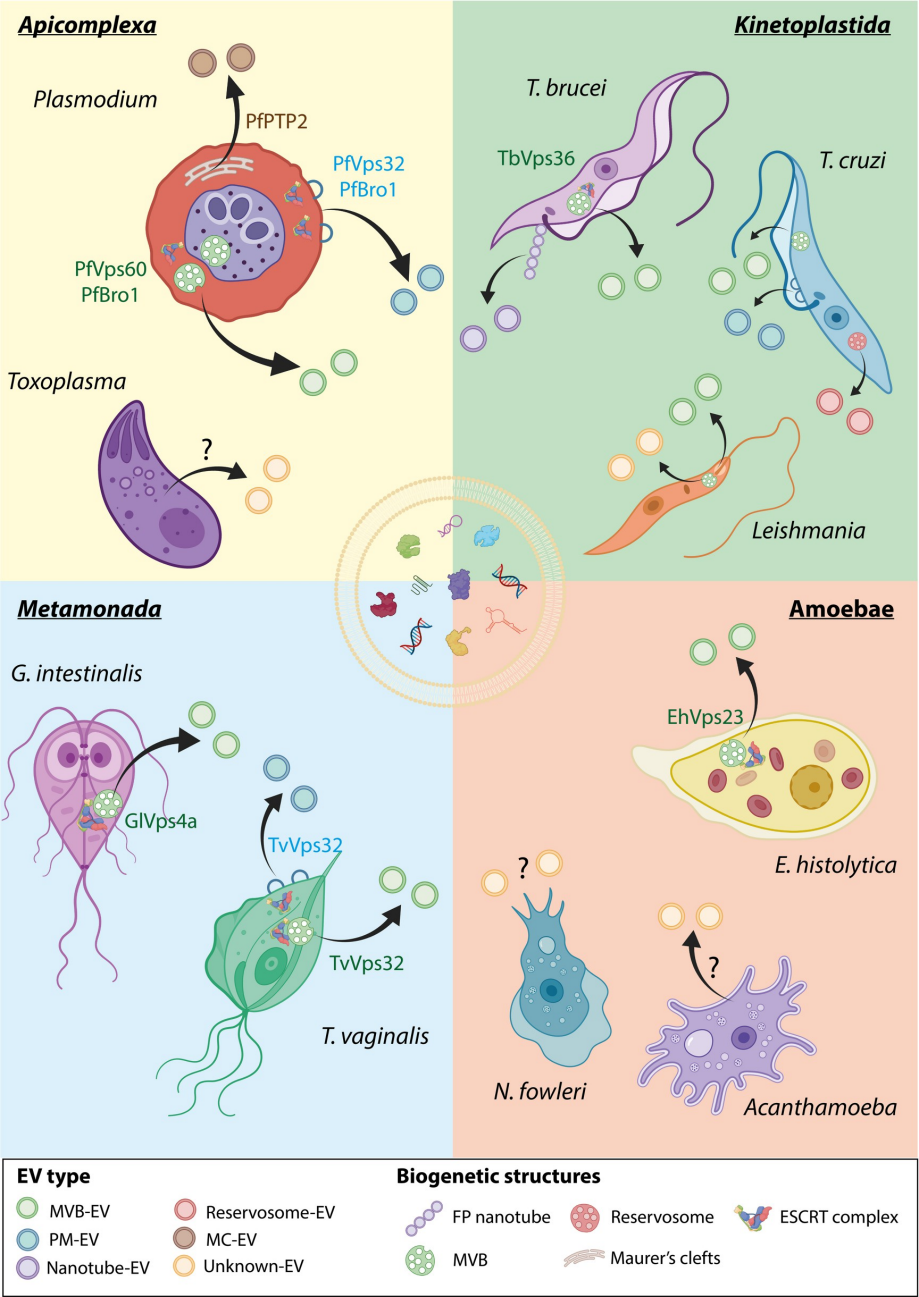
Overview of EV biogenesis and ESCRT protein involvement in human parasitic protozoa. The schematic diagram represents the current knowledge about EV biogenesis mechanisms in the most studied parasitic protozoa, organized by taxonomic and clinical proximity: apicomplexans, kinetoplastids, metamonads, and amoebae. The EVs’ colors reflect their biogenetic origins: MVB-derived exosomes (MVB-EV, **green**), plasma membrane–derived ectosomes (PM-EV, **blue**), nanotube-derived EVs (Nanotube-EV, **purple**), reservosome-derived EVs (Reservosome-EV, **red**), Maurer’s cleft-derived EVs (MC-EV, **brown**), or EVs of unknown biogenetic origin (unknown-EV, **yellow**). The **?** symbol indicates those parasites in which EV secretion is not yet proven, but suggested (*Naegleria fowleri*), or those demonstrated to secrete EVs but their biogenetic origin remains uncertain (*T*. *gondii* and *Acanthamoeba*). The specific ESCRT proteins that have been experimentally demonstrated to participate in each biogenetic route are indicated. **Green letters** represent involvement in the MVB-EV pathway, and **blue letters** represent involvement in the PM-EV pathway, two pathways with proven ESCRT involvement. PfPTP2, a non-ESCRT protein found to be related to EV secretion in *P*. *falciparum* is also presented in **brown letters**. Although *N*. *fowleri* is not taxonomically related to the other amoebae belonging to *Amoebozoa*, it was grouped with the others for the purpose of simplicity. EV, extracellular vesicle; FP, flagellar pocket; MC, Maurer’s clefts; MVB, multivesicular body; PM, plasma membrane. Created with BioRender.com and licensed for publication (agreement number: GK24X52HZC).

A key study improved our understanding of the mechanisms of *P*. *falciparum* EV release by demonstrating the activation of a functional ESCRT-III machinery by an “alternative recruitment pathway,” seemingly independent from other ESCRT subcomplexes [22]. Namely, it was found that two ESCRT-III proteins encoded in the *P*. *falciparum* genome [45], PfVps32 and PfVps60, as well as PfBro1 (a parasitic homologue of the human Alix protein) were all present in EVs derived from *P*. *falciparum*-infected human RBCs but not in EVs derived from naïve RBCs [22]. Although all three proteins localized to the cytoplasm of the parasite, PfVps32 and PfBro1 were also exported to the RBC cytoplasm, where it was suggested that they participate in microvesicle (ectosome) biogenesis and shedding [22] (**Fig 1, *Apicomplexa***). In addition, PfVps60 KO parasites secreted less EVs compared to their *WT* counterparts, lending further support to PfVps60’s participation in EV biogenesis during *P*. *falciparum* infection [22]. The same study used the giant unilamellar vesicle (GUV) membrane model and *P*. *falciparum*-ESCRT-III-purified recombinant proteins to further demonstrate that the PfBro1 protein can trigger the formation of buds by recruiting PfVps32 and PfVps60 proteins to the GUV membrane and activating them [22], as occurs with other ESCRT-III homologues [79,80] during MVB generation (**Fig 1, *Apicomplexa***). Thus, using the purified proteins allowed the recreation of the two mechanisms of EV production in *P*. *falciparum*: MVB generation, as well as membrane shedding in GUVs that mimic the composition of the erythrocyte plasma membrane.

Maurer’s clefts, an elusive membranal system that appears in *P*. *falciparum*-infected RBCs, are also thought to be involved in active EV secretion. This system is critical for the parasite’s ability to actively export PfEMP1, the major virulence factor of malaria, and other parasitic proteins to the RBC membrane [81]. It has been shown that the deletion of PfPTP2, a protein localized to Maurer’s clefts that is involved in transporting PfEMP1 to the RBC membrane [82], leads to a significant reduction in EV secretion from infected RBCs [19] (**Fig 1, *Apicomplexa***).

*T*. *gondii*, too, employs the ESCRT machinery to promote its pathogenesis. It was found that during infection, this parasite recruits three components of the host’s ESCRT machinery [83] to the protein-laden membrane (PVM) that envelopes its parasitophorous vacuole [27]. Two host proteins related to the ESCRT-III machinery, CHMP4B and its regulator, CC2D1A, were highly enriched in the PVM [83]. These findings suggest that host ESCRT machinery promotes or maintains *Toxoplasma*’s nanotubular intravacuolar network [83]. Another study showed that *T*. *gondii* rhoptry neck proteins cooperate to actively recruit different host proteins during invasion, including ESCRT-I components Alix and TSG101 [84]. Parasite mutants that cannot recruit these host proteins showed insufficient invasion into host cells and reduced virulence in mice [84]. In addition, the *T*. *gondii* effector protein TgGRA14 was shown to recruit the host ESCRT machinery to the PV membrane of the parasite and use it for vesicular trafficking and uptake of host cytosolic proteins [85]. Thus, it is evident that *T*. *gondii* is capable of exploiting host ESCRT machinery for resource procurement and vesicular trafficking. These results may also suggest that the parasite uses the host ESCRT machinery for EV biogenesis (**Fig 1, *Apicomplexa***), in line with reports on other intracellular parasites [27]. Further research is needed, however, to uncover the exact mechanism of EV biogenesis in *T*. *gondii* and the players involved.

## III. EV functions and biogenesis in *Kinetoplastida*: *Trypanosoma brucei*, *Trypanosoma cruzi*, and *Leishmania*

Kinetoplastid parasites belonging to the *Trypanosomatidae* family are responsible for a wide variety of human diseases, including leishmaniasis (caused by numerous *Leishmania* species), American Trypanosomiasis or Chagas disease (caused by *T*. *cruzi*), and human African Trypanosomiasis or “sleeping sickness” (caused by *T*. *brucei*) [86]. These three parasitic diseases are considered neglected tropical diseases by the World Health Organization [87] and mainly affect developing countries with impoverished populations [87].

EV secretion is one of the strategies kinetoplastid parasites use to sustain their complex life cycles in the human host [88,89]. These EVs aid trypanosomatids invade into host cells [15,90], communicate with other cells [15,23], advance the course of the disease [91–93], regulate the immune response [92,94,95], and survive in their vectors and hosts [88,89,96]. EVs even possibly play a central role in protein secretion [97–100], as proteins secreted by trypanosomatids, in general, lack a signal peptide; for example, in *Leishmania*, only 5% to 9% of secreted proteins contain a signal peptide [100–102]. Secretome analyses in *Trypanosoma*, *L*. *major*, and *L*. *donovani* revealed that most of the protein secretome in these organisms is released via EVs [97–100].

Most of the current knowledge on trypanosomatid EV biogenesis stems from *T*. *brucei* studies. *T*. *brucei*-derived EVs have been implicated in multiple biological functions relevant to pathogenesis, including antigen and virulence factor transference (mainly of the variant surface glycoproteins) [103], survival of the extracellular parasites in blood [98], host erythrocyte lysis [103], immune response manipulation [104], and central nervous system inflammation [105]. This wide range of cellular targets points towards different EV subpopulations with different possible biogenetic origins. One subpopulation of *T*. *brucei* EVs is generated in the flagellar pocket [98,103] (**Fig 1, *Kinetoplastida***), a main cellular compartment unique to trypanosomatids with central functions in vesicular trafficking, endocytosis, and exocytosis [106]. This EV subgroup range in size from 50 to 100 nm and originate from nanotubes [103], which are highly dynamic filamentous membrane projections of up to 20 μm that bud from the flagellar pocket and vesiculate to form EVs [98,103]. These nanotube-derived EVs were first identified in blood-stage trypomastigotes [103] and later on also in vector-residing procyclic trypomastigotes [23].

*T*. *brucei* is also able to generate exosomes of 40 to 100 nm via MVBs when the RNA *trans-s*plicing pathway is disrupted [23], as well as during heat shock [23]. Despite appearing to be secreted from the flagellar pocket compartment, these exosomes differ morphologically from previously characterized flagellar pocket nanotube-derived EVs [98,103]. MVB-derived EVs were shown to regulate social motility in *T*. *brucei* procyclic parasites, an important process during vector invasion and proliferation [23,107].

*T*. *cruzi*, too, employs EVs for a range of functions, including host cell invasion [15,90,108,109], pathogenesis and disease progression [91,95], vector colonization and metacyclogenesis [15,110], and immune system evasion and manipulation [111–113]. *T*. *cruzi*-derived EVs are considered regulators of both the acute and chronic forms of Chagas disease [114], achieved through the modulation of inflammation and remote signaling between extracellular trypomastigotes and intracellular amastigotes in diverse host cell niches [114]. EVs have been successfully isolated from most stages of the *T*. *cruzi* life cycle [115], including the vector-invasive epimastigote [110,116], axenic amastigotes [117], trypomastigotes [90,91,116] (bloodstream, tissue culture-derived, and metacyclic), and *T*. *cruzi*-infected host cells [118], and their cargo has been compared across stages [116] and strains [111].

Proteomic analysis of the *T*. *cruzi* secretome revealed that epimastigotes and metacyclic trypomastigotes both secrete two subpopulations of EVs: larger ectosomes that bud directly from the cell membrane surrounding the flagellum and smaller exosomes, possibly MVB-derived [119], which are also released through the flagellar pocket [119] (**Fig 1, *Kinetoplastida***). These data suggest that, similar to the case of *T*. *brucei* [23], there may be at least two EV biogenesis pathways in play in *T*. *cruzi*: plasma membrane–derived ectosomes and MVB-derived exosomes [119], both in the flagellar pocket.

Just like in *T*. *brucei* and *T*. *cruzi*, the EVs secreted by *Leishmania* play essential roles for its pathogenesis, including immune cell regulation [92,120,121], invasion and intracellular proliferation [122], cutaneous lesion formation [123], immune cell recruitment [124], and disease progression [125,126]. EVs have been isolated from most stages of the life cycle of the *Leishmania* parasite, including axenic amastigotes [16], intracellular amastigotes in infected macrophages [92–94], procyclic and metacyclic promastigotes [120,123,127], indicating that both its intracellular and extracellular forms produce EVs. It was further determined that EVs that originate from amastigotes and promastigotes have different sizes [120]. The proteomic profile of procyclic and metacyclic promastigotes is also unique [127], suggesting these stages might have specific biogenetic pathways that generate EVs of diverse cargos and sizes.

Unlike *Trypanosoma*, the flagellar pocket in *Leishmania* has not been demonstrated to be directly involved in EV secretion, although the protein secretion endosomal systems are also located in the flagellar pocket [128,129]. Moreover, it was found that the LRV1 virus, which infects *L*. *guyanensis*, is released within the parasite’s EVs [130]. The virus also exploits the parasite’s biogenetic machinery in order to be loaded onto EVs [130]. LRV1 virions were enriched both in MVBs and the flagellar pocket of the parasite [130], suggesting that the flagellar pocket may be involved in EV biogenesis also in *Leishmania* [130] (**Fig 1, *Kinetoplastida***). Whether the flagellar pocket and MVBs are responsible for EV release in conditions other than LRV1 infection and whether the ESCRT system is associated with these organelles in the context of EV biogenesis remain elusive questions.

Little is currently known about the EV biogenesis pathways in kinetoplastid parasites [88,126]. Yet, at least in the case of in *T*. *cruzi*, multiple stress conditions, such as nutrient starvation [15], low temperature and pH [131], nitrosative stress [131], and challenges with host cells [95], trigger EV release. In *Leishmania*, changes in extracellular temperature and pH can trigger EV secretion too [100]. This points toward EV release as being a tightly regulated process in these parasites, with different biogenetic pathways possibly activated by different stressors.

A phylogenetic study of the ESCRT system identified several homologues of ESCRT-I, -II, and -III family members in *T*. *brucei*, *L*. *major*, and *T*. *cruzi* [45], indicating that this system is conserved in *Kinetoplastida* [45]. The finding of protein homologues of most of the ESCRT families in kinetoplastids suggests that the ESCRT complex is fully functional in these organisms and can be active in both extracellular and intracellular stages of infection, potentially freeing them from total dependence on the host-derived ESCRT complex [27]. Kinetoplastid ESCRT protein members were shown to be involved in canonical functions such as endocytosis [46], ubiquitin-tagged protein degradation [45,132], surface protein recycling, and vesicular traffic to the endosome [45,46,133], and were suggested to be involved also in EV biogenesis [23,88,126].

Four ESCRT proteins have been fully characterized in *T*. *brucei* (TbVps23, TbVps28, TbVps4, and TbVps24) and demonstrated to be important players in endocytosis of ubiquitinated proteins [45], lysosomal degradation, and recycling of the Invariant Surface Glycoprotein 65 (ISG65) [46,133], all of which occur within the flagellar pocket. A link between MVBs and the flagellar pocket in *T*. *brucei* arises from the finding that the flagellar pocket is involved in the rescue of variant and invariant surface glycoproteins internalized in MVBs [132,134] and targeted to the lysosome by recycling back to the membrane [132,134]. This evidence may suggest an intimate connection between ESCRT proteins in the *T*. *brucei* endosomal compartment, MVBs, and the flagellar pocket. Nevertheless, the ESCRT machinery has yet to be directly associated with flagellar pocket EV biogenesis.

Only the ESCRT-II component Vps36 has been shown to be critical for the secretion of MVB-derived EVs [23] in *T*. *brucei*. Specifically, silencing Vps36 hindered the production of EVs derived from MVBs but did not stop the secretion of nanotube-derived EVs [23]. Thus, the biogenesis of nanotube-derived EVs in the *T*. *brucei* flagellar pocket may be independent from the ESCRT complex, or at least not involve Vps36 [23], while MVB-derived EV biogenesis and secretion does rely on Vps36 [23] (**Fig 1, *Kinetoplastida***). It further stands to reason that some of the yet understudied ESCRT candidates may be involved in flagellar pocket EV biogenesis through similar mechanisms. This initial evidence links ESCRT proteins to EV biogenesis in trypanosomatids and opens the door to future explorations of the specific roles and involvement of ESCRT proteins in *T*. *brucei*.

In *T*. *cruzi*, the EV biogenetic pathways remain almost completely unknown, and no ESCRT proteins have been characterized yet, despite their predicted existence [45]. Similarly, only a few studies have investigated the biogenesis of EVs in *Leishmania*. Analogues of the ESCRT complex families I, II, and III were found in *Leishmania* [45], but their roles in EV biogenesis remain unexplored. EV proteomic studies have found Rab GTPases [100], Alix [100], and other ESCRT homologues to be present within *Leishmania* EVs [99–101,123]. TEM analysis has identified MVBs, suggested to be active in EV secretion, in *Leishmania* promastigotes within the vector sandfly [123]. Collectively, these data suggest that MVBs and the ESCRT system might be active in EV biogenesis, at least in the extracellular stages of this parasite. Interestingly, human ESCRT member proteins have been identified in EVs secreted by *Leishmania*-infected macrophages [92]. Thus, it has been suggested that intracellular *Leishmania* amastigotes may also be able to “hijack,” at least partially, the host cell’s ESCRT system and vesicle secretion mechanisms to release their own EVs [27,100]. Therefore, *Leishmania* could export virulence factors into the host’s cytosol or to the parasitophorous vacuole and “hijack” the existing EV protein sorting and secretion machinery of its host cells.

Importantly, both *T*. *cruzi*’s and *T*. *brucei*’s EV production has been related to small RNA pathways. For example, both tsRNA [15] and SL RNAs [23] are recruited to MVBs and endosomal compartments and sorted into EVs during stress. The sorting of specific tRNA- and rRNA-derived small RNA into EVs was also demonstrated in *Leishmania* [16]. In *T*. *cruzi*, nutrient deprivation causes tsRNA to be recruited to endosomal compartments identified as reservosomes [15] and then released within EVs of 20 to 200 nm in size [15]. Reservosomes may represent a distinct EV biogenetic pathway [15,135] (**Fig 1, *Kinetoplastida***), as they are enriched in proteins that are also abundant in EVs, such as cruzipain and Rab homologues [89,136]. EVs released during nutritional stress of *T*. *cruzi* epimastigotes were shown to contain unique small RNAs derived from rRNA, tRNA, sno/snRNAs, and protein coding sequences [135]. Collectively, these data suggest that specialized EV-RNA sorting pathways may exist in these parasites as part of their EV biogenetic machinery. However, further research is needed to establish EV biogenetic pathways.

## IV. EV functions and biogenesis in *Metamonada* flagellates: *Trichomonas vaginalis* and *Giardia intestinalis*

The large group of Metamonad protozoans include the retortamonads, diplomonads, and, possibly, the parabasalids and oxymonads as well [137], as the composition of this group is not entirely settled and taxonomic classification has been revised several times. Among its members are several human pathogens, such as *G*. *intestinalis* and *T*. *vaginalis* [138]. *T*. *vaginalis* is the causative agent of trichomoniasis, the most common pathogenic protozoan infection worldwide [139], with an estimated 160 million new cases of infection each year [139]. As to *G*. *intestinalis* (syn. *Giardia lamblia*), it is probably the most notorious of all metamonads [140], causing giardiasis, a gastrointestinal disease of humans and animals [140], with approximately 280 million new human cases of infection annually [138].

Metamonads utilize EVs (both exosomes and ectosomes) as a mechanism for intercellular communication [138,141,142], for survival and persistence within their hosts [143], and for host manipulation [138]. Among the wide range of bioactive molecules transferred within the various EV types [143] are virulence and differentiation factors [138]. The exosomes and microvesicles [143] that *T*. *vaginalis* secretes, for example, have been implicated in the modulation of parasite adherence [144,145] and the delivery of virulence factors to host cells [144]. They were also shown to modulate its host’s immune response by manipulating the expression of cytokines IL6 and IL8 in host vaginal epithelial cells [144] and modulating macrophage activity (by increasing the release of NO and inducing the secretion of anti-inflammatory cytokine IL10) [146].

*Giardia* parasites contain several acidified peripheral vacuoles (APVs), found adjacent to the plasma membrane, which act partially as endosomes and lysosomes [147,148]. Using high-resolution electron microscopy, it was found that ILVs are present inside some of the APVs, suggesting that those organelles could also act as MVBs [148]. Two distinct populations of EVs [24,149], each with a distinct proteomic profile and size, were described: exosome-like vesicles (ElVs) and microvesicles [24,149]. Both subtypes are internalized by mammalian cells [149], and one of them is able to mildly activate immature dendritic cells [150]. In addition, *Giardia* EVs are also involved in disrupting host intestinal epithelial junctions and in inhibiting the growth of commensal bacteria [151], which may account for the prevalence of post-infectious syndromes following disease eradication [151,152].

Several studies provide evidence for the involvement of ESCRT machinery in EV biogenesis in both *T*. *vaginalis and G*. *intestinalis* (and also cargo sorting in the case of *T*. *vaginalis*; [145]). Proteomic analysis of isolated exosomes and ectosomes harvested from *T*. *vaginalis* identified ESCRT-III machinery member VPS32 [143,144]. Indeed, VPS32 plays a key role in EV biogenesis and cargo sorting in *T*. *vaginalis* [145] (**Fig 1, *Metamonada***). By using transgenic parasites expressing a standard *T*. *vaginalis* expression vector, VPS32 was found to localize to ILVs inside the MVBs as well as to ectosomes that protrude from the cell surface [145] **(Fig 1, *Metamonada*)**. VPS32 is also present in EVs being endocytosed to the cell or exocytosed out of the cell [145]. Transgenic parasites overexpressing VPS32 adhered more strongly to host prostate cells compared to control parasites, highlighting this protein’s important role in mediating parasite–host interactions [145]. Two proteins identified in the proteomics data are Rab5 and Rab7, both of which have been implicated in exosome release in other cell types [153,154].

*Giardia* possess a relatively reduced portfolio of ESCRT machinery, containing only orthologs for Vps22 and Vps25 (ESCRT-II proteins) [155], Vps2 and Vps24 (ESCRT-III proteins) [45,155], Vps46 and AAA-ATPase Vps4 in its genome [45,155]. Transgenic parasites expressing deficient GlVps4a protein demonstrate a reduction in ILV formation on the APV and decreased ElV release compared to *WT* parasites [24] (**Fig 1, *Metamonada***). Moreover, overexpression of GlVps4a led to more ILVs, but the amount of produced ElVs was similar to that of the *WT* strain, suggesting the involvement of another mechanism in EV release, independent of GlVps4a [24]. GIRab11, too, is involved in EV production in *G*. *intestinalis* [24]. Down-regulation of GIRab11 was shown to inhibit ElV formation in Rab11-deficient transgenic cells, while its overexpression raised the number of APVs containing ILVs, but not ElV release, suggesting that it participates in the biogenesis of ILVs [24]. Lastly, adding exogenous ceramide caused an increase in ILV formation inside APVs, and its localization in the endoplasmic reticulum, APV, and ILV membranes [24], similar to a known ESCRT-independent ceramide pathway involved in ILV and exosome formation [156]. In conclusion, ESCRT, Rab, and ceramide play a role in EV biogenesis in *G*. *intestinalis*.

## V. EV functions and biogenesis in amoebae

Amoebae encompass a heterogeneous group of protozoa that move, at least in one phase of their life cycle, through cytoplasmic projections [157]. These organisms were grouped under the classical, yet obsolete, taxonomic group *Sarcodina* [157]. The biology of amoebae is quite variable, ranging from free-living organisms to facultative and obligatory parasites [157,158]. Thus, modern taxonomic approaches have reclassified many of its members to diverse taxons [158]. Five amoeba parasite species encompass most of the clinical cases in immunocompetent humans: *Entamoeba histolytica* (causes intestinal amoebiasis) [159], *Naegleria fowleri* (causes primary amoebic meningoencephalitis) [5], *Balamuthia mandrillaris* (causes granulomatous amoebic encephalitis), *Acanthamoeba* spp. (causes amoebic keratitis), and *Sappinia diploidea* (causes nongranulomatous amoebic encephalitis) [5].

The most studied amoeba-derived EVs are those produced by *Acanthamoeba castellanii* [160,161], and their immunomodulatory effects were explored in macrophages [162]. EV production by *N*. *fowleri* has not been demonstrated yet, even though it was shown to produce membrane vesiculation as a complement resistance mechanism [163] and to feature contact-dependent release of electrodense vesicles [164]. Moreover, several pathogenicity factors of *N*. *fowleri* are stored in intracellular vesicles, such as naegleriapore A and B [165], or appear in membrane-derived vesicles, such as the virulence factor CD59-like protein [166]. These data suggest the involvement of vesicular trafficking and, possibly, EV secretion, in the pathogenesis of *N*. *fowleri*, a parasite well-known to possess multiple pathogenic tools such as amoebostomes, secreted cytolytic enzymes, and immune protein deactivators [167]. EVs secreted by *Entamoeba histolytica* were characterized by proteomics [168], and specific small RNA populations were identified [168]. These EVs were demonstrated to regulate parasite–parasite communication during encystation [168]: While EVs derived from cysts promoted encystation of active trophozoites, trophozoite-derived EVs prevented encystation [168].

EV biogenesis remains mostly an open question in amoebae (**Fig 1, Amoebae**). ESCRT I, II, III, and ESCRT-associated homologues were identified in *Naegleria gruberi* [45], a nonpathogenic amoeba closely related to *N*. *fowleri*. Additionally, the amoebic homologue of Alix, an associated ESCRT protein, was found to be up-regulated in pathogenic strains of *N*. *fowleri* [169]. Members of ESCRT families 0, I, II, III, and accessory proteins were demonstrated to be up-regulated during erythrophagocytosis by *E*. *histolytica* [51]. Follow-up studies characterized the roles of the following members of the ESCRT system in *E*. *histolytica*: EhVps4 [51] (ESCRT accessory protein), EhADH112 [50] (homologue of human Alix), EhVps20, EhVps24, EhVps32, EhVps2 [48,50] (ESCRT-III members), and EhVps23 [47] (ESCRT-I member). These proteins were shown to be fundamental in canonical ESCRT activities, such as phagocytosis [47,49], MVB formation [50], and ubiquitin-mediated vesicular trafficking [47–49]. Importantly, while EV biogenesis was not directly assessed, ESCRT-I member EhVps23 was observed within MVBs as well as in secreted exosomes [49,168], suggesting its connection to exosome biogenesis in *E*. *histolytica* (**Fig 1, Amoebae**). Interestingly, trophozoites overexpressing EhVps23 presented increased growth, phagocytosis, migration, and in vivo hepatic amoebic abscess formation [49], suggesting that this protein serves as an important pathogenesis factor [49]. Collectively, these data point to the involvement of ESCRT proteins in *E*. *histolytica* vesicular trafficking and, potentially, EV secretion, but further studies are needed to determine the involvement of the ESCRT system.

## VI. Concluding remarks

The role of EVs in human parasitic protozoan biology and pathogenesis has been demonstrated by a variety of studies for apicomplexans, trypanosomatids, metamonads, and amoebae. In this review, we postulate that the ESCRT protein system, which is widely conserved throughout the evolution of protozoans [45], is an intimately involved player in EV biogenesis in these parasitic pathogens. ESCRT homologues have been identified and studied in all of the clades of human parasitic pathogens, with several studies directly characterizing the involvement of ESCRT protein members in EV biogenesis, specifically in *Plasmodium falciparum* [22], *Trypanosoma brucei* [23], *Trichomonas vaginalis* [145], *Giardia intestinalis* [24], and *Entamoeba histolytica* [49]. In addition, host- and parasite-derived ESCRT proteins are commonly found in EV proteomic profiles, and functional MVBs with ILVs have been observed multiple times in diverse parasites. Alternative (and possibly complimentary) EV biogenetic routes independent from the ESCRT system may be active and central in protozoan parasites, as was indeed shown for *P*. *falciparum* [19], *Trypanosoma* [15,23], and *Giardia* [24]. Some intracellular parasites may even be able to “hijack” the host’s ESCRT system for their own EV secretion [27], as viruses do [34,36].

The wide diversity in EV biogenesis throughout protozoan parasites is coherent with the different EV subpopulations recently identified in several parasites [23,63]. There is also great diversity in the size and protein cargo among the different life cycle stages and environmental stress conditions. Namely, parasites may use diverse EV biogenesis pathways as a robust response to environmental cues, including parasite–parasite and host–parasite interactions.

**Fig 2:**
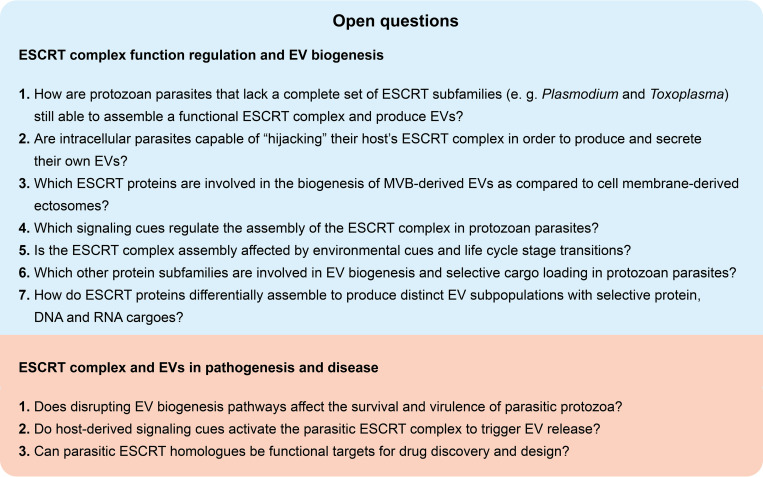
Open questions in EV biogenesis of parasitic protozoa. ESCRT, Endosomal Sorting Complex Required for Transport; EV, extracellular vesicle; MVB, multivesicular body.

Two important obstacles that research into parasitic protozoa EV biogenesis faces are (**1**) the huge biological divergence of parasitic protozoa, which hinders the ability to find protein homologues throughout the widely diverse parasitic clades, and (**2**) the limited availability of experimental research models and genetic tools for many parasites, especially for intracellular stages, which makes the study of parasitic EVs methodologically challenging. Additionally, most mechanistic studies in the field have been performed with EVs isolated from in vitro parasite cultures, since their isolation and characterization from clinical in vivo samples remains arduous. Future studies that adapt more powerful bioinformatic tools for phylogenetic analyses of protein and gene homology, as well as the development and application of reproducible gene editing techniques to protozoans and improved EV isolation methods from clinical samples, will undoubtedly advance the field toward the full elucidation of the biological roles of understudied proteins in EV biology. Some current open questions are suggested in the **Fig 2. Open questions in EV biogenesis of parasitic protozoa** box.
